# Helicobacter pylori Thioredoxin1 May Play a Highly Pathogenic Role via the IL6/STAT3 Pathway

**DOI:** 10.1155/2022/3175935

**Published:** 2022-08-01

**Authors:** Yanlei Guo, Shigang Ding

**Affiliations:** Department of Gastroenterology, Peking University Third Hospital, Beijing 100191, China

## Abstract

**Background:**

Recent studies have shown that CagA is considered highly pathogenic to helicobacter pylori (HP) in Western populations. However, in East Asia, CagA positive HP can be up to 90%, but not all patients will lead to gastric cancer. Our research group has found that HP thioredoxin1 (Trx1) may be a marker of high pathogenicity. Here, we investigate whether HP Trx1 exerts high pathogenicity and its internal molecular mechanism.

**Materials and Methods:**

We constructed the coculture system of high-Trx1 HP and low-Trx1 HP strains with gastric epithelial cell lines separately and detected the influence of HP strains. The cells were stained by AM/PI, and the cell's mortality was assessed by fluorescence microscope. The cell's supernatants or precipitates were collected to detect the expression of IL6. In addition, the cell's precipitates were collected, and the expression of p-STAT3 was detected by western blot. Furthermore, the cell's supernatants were collected for detecting the expression of 8-OHDG to investigate the extent of DNA damage.

**Results:**

The high-Trx1 HP can cause higher mortality of GES-1 cells compared with the low-Trx1 HP group (high-Trx1 HP (4.53 ± 0.56) %, low-Trx1 HP (0.39 ± 0.10) %, *P* < 0.001). The mRNA and protein level of IL-6 in AGS and GES-1 cells were increased during HP infection, and the expression of IL-6 in the High-Trx1 HP group was much higher than the low-Trx1 HP group. Besides, the expression of p-STAT3 was higher in the HP-positive gastric mucosa. And the expression of p-STAT3 in the high-Trx1 HP group was significantly upregulated compared with the low-Trx1 HP group. Furthermore, the expression of 8-OHDG in the high-Trx1 group was much higher than the low-Trx1 group (high-Trx1 HP (5.47 ± 1.73) ng/ml, low-Trx1 HP (2.89 ± 1.72) ng/ml, *P* < 0.05).

**Conclusion:**

HP Trx1 may play as a marker of high pathogenicity, and the high-Trx1 HP could mediate the pathogenic process of HP infection via the IL6/STAT3 pathway.

## 1. Introduction

Gastric cancer is a worldwide commonly diagnosed malignant tumor with a high mortality rate in recent years. And the WHO has indicated Helicobacter pylori (HP) as the class I carcinogen of gastric cancer in the year 1994 [1]. HP is a spiral-shaped, gram-negative bacterium pathogen that colonizes approximately 50% of the world's human population [2]. In addition, the morbidity and mortality of gastric cancer in China are at the forefront of malignant tumors, and the infection rate of Helicobacter pylori (HP) in China is high, with an average of 54.76% [3].

In recent years, the relationship between HP and gastric cancer has received increasing attention. However, the specific pathogenic mechanism of HP remains unclear. The HP strains carrying with CagA are at higher risk of developing into gastric carcinoma and have worse clinical outcomes [4]. Studies have shown that CagA and VacA are considered to be highly pathogenic to HP in Western populations. However, in East Asia including China, the CagA and VacA positive HP can be as high as 90%, but not all patients infected with this HP will lead to gastric cancer, so it is speculated that there may be other virulence factors that play an important role [5]. Individuals infected with highly pathogenic HP are considered to be at higher risk of suffering from gastric cancer. Therefore, early screening and targeted eradication treatment according to the pathogenicity of HP are of great significance to the prevention of gastric cancer.

Thioredoxin (Trx) is a small molecule protein widely existing in the human body and bacteria, which can be divided into two types, Trx1 and Trx2. Trx1 has a highly conserved sequence “Cys-Gly-Pro-Cys,” hich carries disulfide bonds and dithiol groups with redox function [6]. Trx1is a redox protein that plays an important role in protecting HP bacteria, promoting their long-term colonization, and leading to gastric cancer [7]. Our previous research group has found that HP with high expression of Trx1 might be highly pathogenic, and it was studied in histology, cytology, and animal experiments. It was found that the expression of Trx1 in HP bacteria isolated and cultured from gastric mucosa of patients with gastric cancer was significantly higher than that of patients with peptic ulcer or gastritis, which suggested for the first time that HP Trx might be related to the carcinogenesis mechanism [8]. In addition, the results suggested that High-Trx1 HP can have a stronger influence on the gastric epithelial cell line, in which HP Trx1 can promote the apoptosis of human normal gastric epithelial cell line GES-1 and the proliferation of human gastric cancer cell line BGC823, and the balance between proliferation and apoptosis of gastric epithelial cell line is disturbed, which further suggested that HP Trx1 may promote the occurrence and development of gastric cancer [8]. Furthermore, we constructed a model of gastric cancer infected by the low-Trx1 HP or the high-Trx1 HP in Mongolian gerbils. It was found that in the High-Trx1 HP group, the gastric mucosa lesions of Mongolian gerbils were more serious, and the incidence of gastric cancer was higher, which further suggested that the high-Trx1 HP might have stronger pathogenicity [9]. To conclude, our previous research group inferred that Trx1 may be a marker of HP with higher pathogenicity; however, the specific mechanism needs further study.

During HP infection, the expression of IL6 in the gastric mucosa was upregulated, and IL6 can promote the activation of signal transducer and activator of transcription 3 (STAT3), which is closely related to the occurrence, development, and metastasis of malignant tumors [10]. The STAT3 signaling pathway is involved in regulating DNA transcription and gene expression and affects biological processes such as cell proliferation, differentiation, and apoptosis [11]. Besides, the previous studies have shown that the downregulation of STAT3 activity was related to the reduction of DNA damage, to maintain genome stability and protect against cell death [12]. It has been reported that as many as 70% of human tumors have abnormal enhancement of STAT3 activity, including gastric cancer, esophageal cancer, liver cancer, and other tumors [13, 14]. Studies have shown that the phosphorylation level of STAT3 in the gastric mucosa was upregulated during HP infection, and the activation level of STAT3 was related to the occurrence and development of HP-related gastritis and cancer [15].

To sum up, combined with the existing literature reports and the previous research of our group, it is suggested that HP Trx1 may be a marker of high pathogenicity, but the mechanism is unclear yet. Based on the previous work, this study will further explore the correlation between the activation of oncogene STAT3 and DNA damage to further clarify the pathogenicity of HP Trx1 and its potential molecular mechanism.

## 2. Materials and Methods

### 2.1. HP Culture

In this study, we used the following strains: high-Trx1 HP strain, low-Trx1 HP strain, the standard HP ATCC26695 strain, and the CagA-knockout HP ATCC 26695 strain, all of which were preserved in the Key Laboratory for HP infection and upper gastrointestinal diseases in Peking University Third Hospital. The high-Trx1 HP and low-Trx1HP strains were isolated and screened from clinical gastric mucosa samples by our research group. The specific screening steps are as follows: the expression of Trx1mRNA in HP strains was assessed by real-time PCR. Next, by the statistical method, it was found that HP Trx1 was highly expressed when △CT value was less than 8.86, and HP Trx1 was poorly expressed when △CT value was greater than 28.05. Finally, we selected the high-Trx1 and low-Trx1 HP strain. This part of the work has been completed by the members of our research group in the early stage [16]. HP bacteria were cultured on the blood agar plates containing 43 g/L Karmali solid culture medium (OXOID, Basingstoke, UK), 10% sterile defibrinated sheep blood (Landbridge technology, Beijing, China), and Helicobacter pylori selective supplement (OXOID). Besides, the HP was cultured under microaerobic conditions (85%N2, 10%CO2, 5%O2, 37°C).

### 2.2. Cell Culture and HP-Cell Coculture Assays

AGS and GES-1 cells were cultured in RPMI 1640 medium (HyClone, Logan, UT, USA), and the media were supplemented with 10% fetal bovine serum (Gibco, Grand Island, NY, USA) and 100 U/ml penicillin-streptomycin (Gibco). All cells were cultured in a humidified atmosphere at 37°C containing 5% CO2. The HP-cell coculture assays were previously described [17]. Briefly, the bacteria were harvested and washed three times, then resuspended in PBS at a density of 1∗10^8^ CFU/ml. Cells were infected with HP for 24 h at an infection MOI of 100 : 1.

### 2.3. Clinical Samples

The human gastric mucus samples were collected from the tissues of patients undergoing gastroscopy in Peking University Third Hospital. Diagnoses of all samples were confirmed histologically by two independent pathologists, and all tissues were assessed by hematoxylin-eosin staining. All collected HP positive tissues were confirmed by Warthin-Starry staining. This research was approved by the Medical Ethics Committee of the Peking University Third Hospital Institutional Review Board, and written informed consents were signed by all patients.

### 2.4. RNA Isolation and Quantitative Real-Time PCR

Total RNA was extracted from cells or tissues using a trizol reagent (Invitrogen, Carlsbad, CA, USA). The total RNA (1 *μ*g) of every sample was reverse transcribed using the Fast-King RT kit (Tiangen Biotech, Beijing, China). The Bio-Rad iQ5 real-time PCR detection system was used with a Talent qPCR PreMix SYBR Green kit (Tiangen Biotech) according to the manufacturer's instructions. Relative expression of each target gene was determined by normalization to the expression of GAPDH. The primers were synthesized by the Shanghai Sangon company, and the primer sequences were shown in Table 1.

### 2.5. Western Blot Analysis

Cells were harvested with RIPA buffer and incubated on ice for 30 min. The concentration of total protein was quantified by the BCA Protein Assay kit (Applygen, Beijing, China). Equal protein lysates were loaded for each sample and then transferred to polyvinylidene fluoride membranes. The membranes were blocked with 5% BSA for 1 h at room temperature and incubated overnight at 4°C with the following primary antibodies: p-STAT3 antibody1 : 1000 (CST, USA), STAT3 antibody 1 : 1000 (CST, USA), and *β*-actin 1 : 6000 (Applygen, China). The membranes were incubated with anti-rabbit IRDye 800CW secondary antibody 1 : 10000 (LI-COR Biosciences, USA), and the bands were scanned on an Odyssey Infrared Imaging System (LI-COR Bioscience).

### 2.6. Immuno-Histochemical (IHC) Staining

The tissues were used to prepare formalin-fixed paraffin-embedded tissue sections. First, the sections were dewaxed in xylene and rehydrated in a gradient of ethanol. Then, antigen retrieval was performed, and the sections were incubated with p-STAT3 antibody 1 : 200 (CST, USA) overnight at 4°C. The sections were washed with PBS and incubated with an IgG-HRP polymer (ZSGB-BIO, Beijing, China). Lastly, the diaminobenzidine substrate was used for color development.

### 2.7. Double Staining of Living and Dead Cells

Discard the cell culture medium and wash it with 1× Assay Buffer 3 times. Add 1 ml staining solution to the cell culture dish and incubate at 37°C for 30 min. Then, the calcein-AM and PI cell dyes (Yeasen, China) were used to fluorescently label dead cells and living cells to analyze the survival status of cells. Lastly, we observed the cell fluorescence under a fluorescence microscope and collected images randomly.

### 2.8. ELISA Assay

First, collect the cell supernatant and load the samples into the ELISA assay (CST, USA). Then, clean the board and add biotinylated antibody to each well and incubate for 90 min. Then, add streptavidin-HRP to each well and incubate for 30 min. Lastly, add TMB into the well and develop the color for 10-20 min. After the reaction is terminated by a stop solution, the OD reading value at the wavelength of 450 nm was detected with a microplate reader as soon as possible.

### 2.9. Statistical Analysis

Statistical analyses were performed using the SPSS 22.0 software, and the differences between two independent groups were analyzed using Student's *t*-test. For multiple comparisons, a one-way ANOVA analysis followed by the Student–Newman–Keuls (SNK) test was used. *P* < 0.05 was considered statistically significant.

## 3. Results

### 3.1. Identification of the Low-Trx1 and High-Trx1 HP Bacteria Strain

First of all, we identified the HP strains we used in the experiments by means of gram staining, oxidase and urease activity test, and real-time PCR measurement before we started the research.

**Figure 1:**
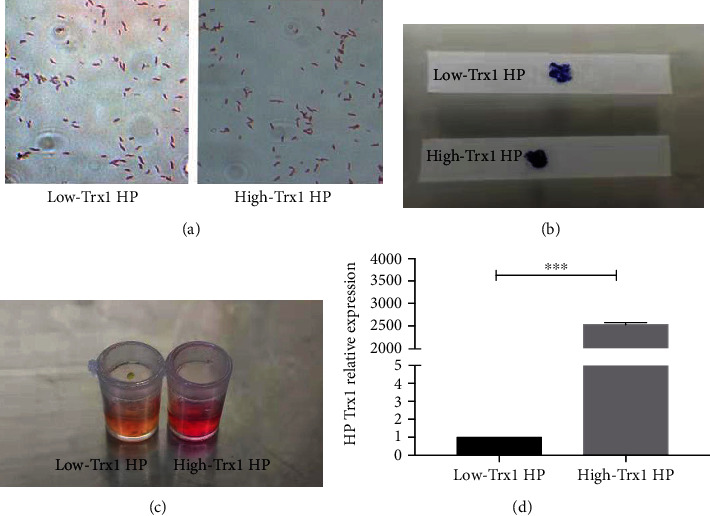
Identification of the low-Trx1 and high-Trx1 HP bacteria strain. (a) Gram staining with low-Trx1 HP and high-Trx1 HP was observed under an oil microscope. (b, c) Detection of oxidase and urease assay with the low-Trx1 HP and high-Trx1 HP. (d) The Trx1 expression of low-Trx1 HP and high-Trx1 HP was analyzed by real-time PCR. Note: low-Trx1 HP means HP with low Trx1 expression, and high-Trx1 HP means HP with high Trx1 expression, ^∗∗∗^*P* < 0.001.

After gram staining, the morphology of the HP strains was observed under an oil microscope. As shown in the picture, gram-negative bacteria with spiral or S-shaped bacteria of different lengths can be seen under the microscope, which are typical HP morphological structures (Figure 1(a)). It is well known that positive oxidase and urease are the characteristics of HP. Consequently, we also detected the oxidase and urease activity of the HP strains. As shown in the result, both groups showed a dark blue reaction at the contact site, which means the oxidase test was positive (Figure 1(b)). And both the reagent in the urease reaction cup turned red, which means the urease test was positive (Figure 1(c)). What is more, we also performed real-time PCR with the HP strains, and the results demonstrated that the expression of Trx1 mRNA in the high-Trx1 HP group was significantly higher than the low-Trx1 HP (Figure 1(d)).

These results indicated that the bacteria strains we used in this research were typical helicobacter pylori strains, and we also identified the Trx1 expression of the low-Trx1 HP and the high-Trx1 HP strains.

### 3.2. The High-Trx1 HP Can Cause Higher Mortality of GES-1 Cells

Our previous research group has found that Trx1 may be a marker of HP pathogenicity. To further explore the pathogenic molecular mechanism of the HP Trx1, we cocultured the HP with GES-1 cells and detected the cells' mortality rate. To observe the different damage effect of low-Trx1 HP and high-Trx1 HP, we stained the GES-1 cells with AM and PI dyes which can mark the dead and live cells separately after HP infection under the fluorescence microscope.

**Figure 2:**
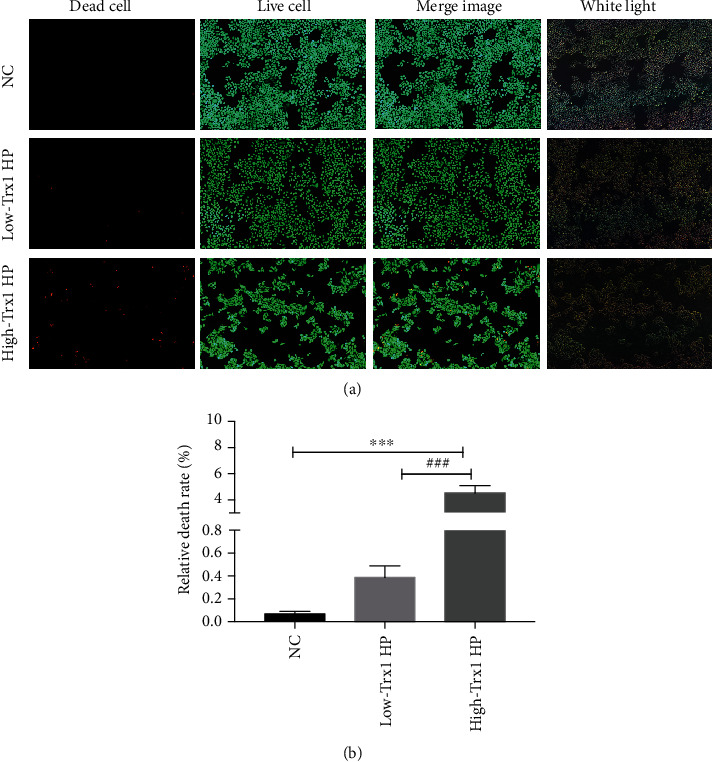
The high-Trx1 HP can cause higher mortality of GES-1 cells. (a) Fluorescence staining of living and dead cells after coculture with low-Trx1 HP or high-Trx1 HP in GES-1 cells. (b) Statistics of dead cells after cocultured with low-Trx1 HP and high-Trx1 HP in GES-1 cells. Note: dead cell means dead cell staining, live cell means live-cell staining, merge image means dead and live cell staining image merging, white light means cell image under bright field, NC means uninfected control group, low-Trx1 HP means HP with low Trx1 expression, and high-Trx1 HP means HP with high Trx1 expression, ^∗∗∗^*P* < 0.001, ^###^*P* < 0.001.

As shown in Figure 2(a), compared to the normal control group, the number of dead cells in both the low-Trx1 HP group and the high-Trx1 HP group were significantly increased. Furthermore, the amount of dead cells in the high-Trx1 HP group was more than in the low-Trx1 HP group. Afterward, we statistically analyzed the data, and as shown in Figure 2(b), the relative cell death rate in both the low-Trx1 HP group and the high-Trx1 HP group was significantly higher than the normal control group. Furthermore, the cell death rate in the high-Trx1 HP group was much more than the low-Trx1 HP group with statistical significance. To conclude, these data indicated that the high-Trx1 HP may be more virulent and result in more cell death.

### 3.3. The High-Trx1 HP Can Cause Higher Expression of IL-6 in AGS and GES-1 Cells

Previous studies have shown that HP infection can promote the expression of inflammatory factors, such as IL-6, which play an important role in the pathogenic process [18]. To explore the cellular inflammatory reaction, we cocultured the high-Trx1 HP and low-Trx1 HP strains with AGS or GES-1 cells separately and collected the cell supernatants and cell precipitates for detecting the expression level of inflammatory factor IL6 by ELISA and real-time PCR.

**Figure 3:**
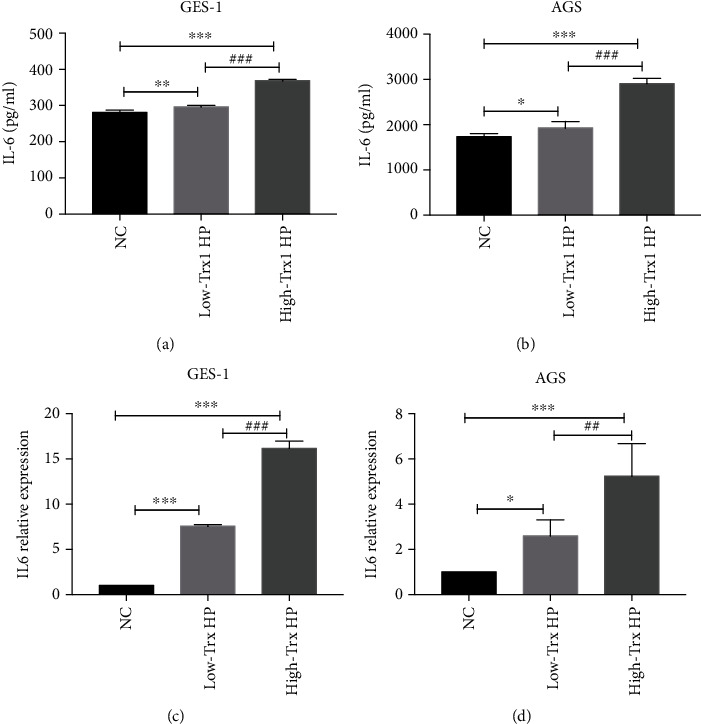
The high-Trx1 HP can cause higher expression of IL-6 in AGS and GES-1 cells. (a, b) The IL-6 expression of GES-1 and AGS cells during low-Trx1 HP and high-Trx1 HP infection were analyzed by ELISA test. (c, d) The IL-6 expression of GES-1 and AGS cells during low-Trx1 HP and high-Trx1 HP infection was analyzed by real-time PCR test. Note: NC means uninfected control group, low-Trx1 HP means HP with low Trx1 expression, and high-Trx1 HP means HP with high Trx1 expression, ^∗^*P* < 0.05, ^∗∗^*P* < 0.01, ^∗∗∗^*P* < 0.001, ^##^*P* < 0.01, and ^###^*P* < 0.001.

As the ELISA assays shown in Figures 3(a) and 3(b), compared to the normal control group, the expression of IL-6 protein in both the low-Trx1 HP group and the high-Trx1 HP group of GES-1 and AGS cells was significantly increased. Moreover, the expression of IL-6 in the high-Trx1 HP group was more than the low-Trx1 HP group both in the GES-1 and AGS cells. Consistently, the IL-6 mRNA levels increased during HP infection, and the expression of IL-6 mRNA in the high-Trx1 HP group was much higher than the low-Trx1 HP group (Figures 3(c) and 3(d)).

These results indicated that the high-Trx1 HP can induce the cell to produce much more IL-6, which may be involved with the pathogenic process of HP infection.

### 3.4. The High-Trx1 HP Can Cause Higher Expression of p-STAT3

**Figure 4:**
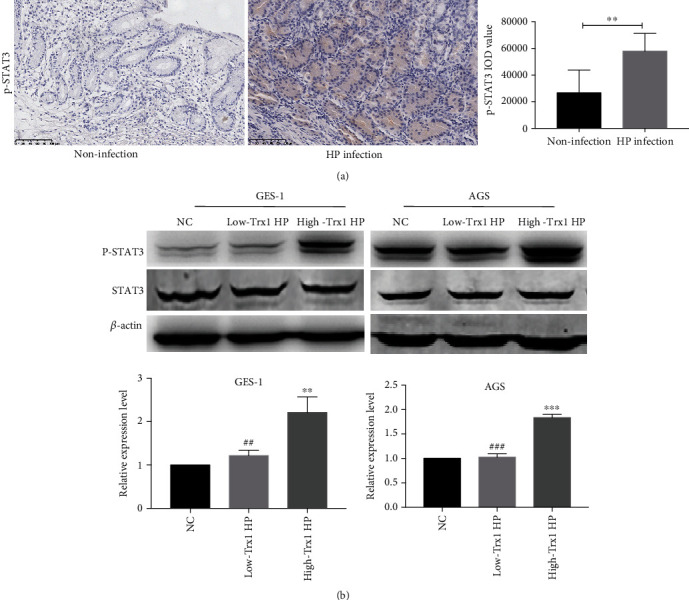
The high-Trx1 HP can cause higher expression of p-STAT3. (a) Representative immuno-histochemical pictures and statistical graph of p-STAT3 in noninfection gastric mucus (*n* = 8) and HP infection gastric mucus (*n* = 8). (b) The p-STAT3 expression of GES-1 and AGS cells during low-Trx1 HP and high-Trx1 HP infection was detected by Western blot. Note: NC means uninfected control group, low-Trx1 HP means HP with low Trx1 expression, and high-Trx1 HP means HP with high Trx1 expression, ^∗∗^*P* < 0.01, ^∗∗∗^*P* < 0.001, ^##^*P* < 0.01, and ^###^*P* < 0.001.

It has long been recognized that IL-6 can activate STAT3 which is closely related to the occurrence, development, and metastasis of malignant tumors during HP infection [10]. To explore the influence of high-Trx1 HP for STAT3 activation, we carried out the following study. First, we collected the HP-negative gastric mucosa samples (*n* = 8) and the HP-positive gastric mucosa samples (*n* = 8) and developed the immunohistochemistry experiment. The results confirmed that the p-STAT3 expression was significantly upregulated in HP-positive gastric mucosa than in the HP-negative gastric mucosa (Figure 4(a)). To verify the influence of HP Trx1 on STAT3, we used GES-1 and AGS cell lines to carry out HP infection experiments in vitro and measured the expression of p-STAT3 by western blot. Consistent with the previous study, we found that the expression of p-STAT3 in both the low-Trx1 HP group and the high-Trx1 HP group of GES-1 and AGS cells were upregulated compared with the normal control group. Besides, we found that in GES-1 and AGS cell lines, the expression level of p-STAT3 during the high-Trx1 HP infection was much higher than the low-Trx1 HP group (Figure 4(b)). Therefore, we speculated the high-Trx1 HP may play a highly pathogenic role via the STAT3 activation.

### 3.5. The High-Trx1 HP Can Cause Much More Serious DNA Damage

Previous studies have shown that the expression of STAT3 was closely related to DNA damage [12]. In the above results, we found that the high-Trx1 HP can induce higher expression of p-STAT3, so we speculated that the high-Trx1 HP may result in stronger DNA damage. To verify the speculation, we studied the changes of 8-OHDG and OGG1 which are related to the degree of DNA damage.

**Figure 5:**
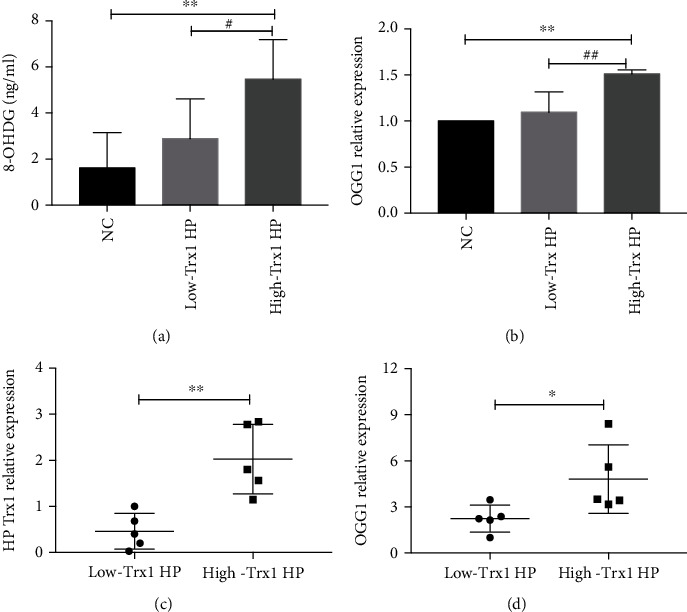
The high-Trx1 HP can cause much more serious DNA damage. (a) The 8-OHDG expression of GES-1 cell during low-Trx1 HP and high-Trx1 HP infection was analyzed by ELISA. (B) The OGG1 expression of GES-1 cell during low-Trx1 HP and high-Trx1 HP infection was analyzed by real-time PCR. (c, d) The expression of HPTrx1 and OGG1 in HP positive gastric mucosa (*n* = 10) was analyzed by real-time PCR. Note: NC means uninfected control group, low-Trx1 HP means HP with lowTrx1 expression, and high-Trx1 HP means HP with high Trx1expression, ^∗^*P* < 0.05, ^∗∗^*P* < 0.01, ^##^*P* < 0.01, and ^###^*P* < 0.001.

In this experiment, we cocultured the GES-1 cell line with the high-Trx1 HP and low-Trx1 HP separately and collected the cell supernatant for ELISA. The results showed that the expression of 8-OHDG in the high-Trx1 group was much higher than in the low-Trx1 group (Figure 5(a)). What is more, we collected cell precipitates for real-time PCR, and the results showed that the expression of OGG1 in the high-Trx1 group was much higher than the low-Trx1 group (Figure 5(b)).

In addition, we divided the gastric mucosa samples of HP-positive gastritis patients into high-Trx1 HP group and low-Trx1 HP group, with 5 cases in each group, according to the real-time PCR results. As the results showed, the expression of OGG1 in the high-Trx1 group was much higher than the low-Trx1 group (Figures 5(c) and 5(d)).

To conclude, we speculated that the high-Trx1 HP may mediate DNA damage by activating STAT3 and then play a highly pathogenic role.

### 3.6. The CagA Gene Cannot Affect the Trx1 Expression in HP

Studies have shown that CagA is considered to be highly pathogenic to HP in Western populations. However, in East Asia including China, CagA may not be directly related to the occurrence of gastric mucosal diseases [5]. In order to verify whether HP Trx can play its pathogenic role independently without the influence of cagA, we conducted the following research.

**Figure 6:**
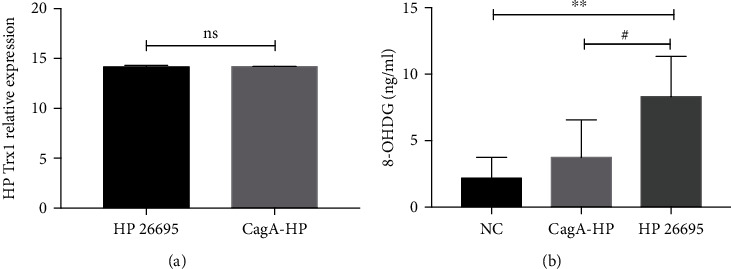
The CagA gene cannot affect the Trx1 expression in HP. (A) The HPTrx1 expression of HP26695 and the CagA knockout HP was analyzed by real-time PCR. (b) The 8-OHDG expression of HP2695 and the CagA knockout HP was analyzed by ELISA. NC means uninfected control group, HP26695 means the Standard HP ATCC26695 strain, and CagA-HP means the CagA-knockout HP ATCC 26695 strain. ns represents nonsignificance, ^∗∗^*P* < 0.01, and ^#^*P* < 0.05.

We measured the Trx1 expression of the Standard HP ATCC26695 strain and the CagA-knockout HP ATCC 26695 strain by real-time PCR, and the result revealed that there was no significant difference in the expression of Trx1 mRNA between the above two HP strains (Figure 6(a)). In addition, we cocultured the GES-1 cell line with the Standard HP ATCC26695 strain and the CagA-knockout HP ATCC 26695 strain and collected the cell supernatant for ELISA. The results showed that the expression of 8-OHDG in the HP ATCC26695 group was much higher than the CagA-knockout HP ATCC 26695 group (Figure 6(b)).

Therefore, it is inferred that HP CagA may have no influence on HP Trx1, and they could play the pathogenic role independently.

## 4. Discussion

At present, studies have made clear the close relationship between HP infection and gastric mucosal diseases, but the pathogenesis of HP has not been fully clarified. Our previous research suggests that Trx1 may be a marker of HP with high pathogenicity and play an important role in the pathogenesis of HP infection. In the present study, we found that HPTrx1 can stimulate gastric cells to produce much more IL6 accompanied by the oncogene STAT3 activation, which may be involved with the highly pathogenic.

Thioredoxin and glutathione systems are the main antioxidant systems. The HP strains do not carry a glutathione system, so the thioredoxin system is essential for HP to survive in an aerobic environment [19]. Organisms are equipped with various thiol-dependent antioxidant systems, which can coordinate the removal of active oxygen and active nitrogen substances [6]. Trx is ubiquitous in bacteria, but there is no glutathione system in many gram-positive bacteria and some gram-negative bacteria, such as HP [20]. Trx system has different meanings in protecting cells from the oxidative stress of different organisms. In mammalian cells, thioredoxin and glutathione systems can cross-supply electrons and act as backup systems for each other [21, 22]. However, the glutathione system does not exist in some bacteria such as HP and E. coli, which makes Trx system vital to the survival of bacteria under oxidative stress [6, 23, 24]. There are obvious differences in the structure and reaction mechanism of Trx receptor between bacteria and mammals, which makes Trx receptor a new target of antibiotics and provides an opportunity to kill bacteria by targeting Trx receptor [25]. The latest research found that ebselen, a stroke drug, is an inhibitor of Trx receptor, which can inhibit the growth of bacteria lacking the glutathione system, providing a new idea for the diagnosis and treatment of glutathione negative pathogens [25, 26]. There is no glutathione system in HP bacteria, and its antioxidation depends on Trx system. At the same time, our research found that Trx1 may be a marker of HP with high pathogenicity. If the action of HP Trx1 can be inhibited, it may provide a new target for the treatment of HP-related gastric mucosa diseases in the future.

8-OHDG is an oxidative adduct produced by reactive oxygen species attacking the 8th carbon atom of guanine base in DNA molecules, which can be used as a biomarker of DNA oxidative damage [27]. DNA damage can be detected in the gastric mucosa during HP infection [17, 28]. Our previous study found that the high-Trx1 HP was related to stronger cellular stress and redox activity-related proteins [29, 30]. Consistent with the previous studies, we found that HP with high expression of Trx1 can produce much more 8-OHDG, suggesting that 8-OHDG may be one of the pathogenic mechanisms of HP Trx1. At the same time, after DNA damage, it can be repaired by the base cleavage enzyme OGG1 [31]. Our results also showed that the expression of OGG1 is higher after coculture with the high-Trx1 HP, which is presumed to be due to the increased expression of 8-OHDG after DNA damage which further suggests that the HP Trx1may play a pathogenic role through the mechanism of DNA damage.

The mechanism of carcinogenesis by HP is complex, including the activation of oncogenes by HP. STAT3 can promote the growth of tumor cells and play an important role in the occurrence and development of cancer. The abnormal expression of p-STAT3 can be found in nearly 70% of cancers, and it can be used as a marker of poor tumor prognosis [32]. STAT3 belongs to the STATs family, and its members include STAT1, 2, 3, 4, 5a, 5b, and 6. When STAT3 is phosphorylated, it gets into the nucleus to combine with the target gene and promotes its transcription, which plays a key role in signal transduction and transcription activation [33]. Sekikawa and others found that STAT3 can play a role in inhibiting the apoptosis of gastric cancer cells, and the overactivation of STAT3 is closely related to the progression of gastric cancer [34]. After coculture of HP and gastric epithelial cell line, the phosphorylation level of STAT3 increased [35].

The previous study has shown that the STAT3's activity is mediated by posttranslational modifications including phosphorylation, acetylation, and reduction/oxidation (redox) processes [36]. And in Busker's study, they used the chemical biology approach to inhibit the function of Trx1, which induced the STAT3 oxidized and prevented the transcriptional activation of STAT3 [37]. In addition, Kim et al. found that auranofin which acts as a potent and specific inhibitor of mitochondrial thioredoxin reductase can block interleukin-6 signal by inhibiting phosphorylation of JAK1 and STAT3 [38]. At present, there are many studies on human Trx1 and STAT3; however, the researches on the Trx1 secreted by HP and STAT3 are still inadequate. Similar to human Trx1, the HP Trx1 possesses the same redox functional domain “Cys-Gly-Pro-Cys,” so it is speculated that there may be a similar molecular mechanism for the HP Trx1 which still needs further exploration [39, 40].

Consistent with previous research results, we found that the expression of p-STAT3 in HP-positive gastric mucosa was higher, suggesting that p-STAT3 may play an important role in HP pathogenesis. Then, we cocultured the gastric cells with high-Trx1 HP and low-Trx1 HP separately and found that the expression of p-STAT3 in cells was much higher after being cocultured with the high-Trx1 HP. Therefore, we speculated that HP Trx1 might play a pathogenic role by regulating the STAT3 gene, thus increasing the risk of gastric carcinogenesis. In addition, Yu et al. found that the inflammatory factor IL6 can promote the phosphorylation of STAT3 and participate in the inflammatory process by regulating downstream gene products and then inducing tumorigenesis [41]. In agreement with previous studies, we found that the expression of IL6 and p-STAT3 in gastric cells was higher after coculture with the High-Trx1 HP. Therefore, we speculate that the high-Trx1 HP may play a pathogenic role through the IL6/STAT3 pathway, but its specific mechanism and downstream molecules of STAT3 need further study.

The carcinogenic effect of HP depends on virulence factors. Current studies have shown that CagA may be a marker of HP with higher virulence, but not all CagA-positive HP strains have a carcinogenic effect, and other pathogenic factors of HP need further research. CagA is a virulence factor of HP which has been widely studied. In this study, we detected the expression of Trx1 in HP strains after the CagA gene was knockout and found that the expression of Trx1 was not affected, which suggested that Trx1 may play a pathogenic role without being affected by CagA. After the standard strain HP ATCC26695 and the CagA gene knockout HP strain were cocultured with the gastric cell, we detected the expression level of 8-OHDG, which showed that the expression of 8-OHDG was lower and the DNA damage level caused by HP became weaker after the CagA gene was knockout, suggesting that CagA gene may play a pathogenic role. Therefore, it is speculated that both Trx1 and CagA may be the key molecules affecting the pathogenesis of HP independently, and further research is needed.

## 5. Conclusion

In summary, this study mainly explored the role of Trx1 in the pathogenesis of HP and its potential molecular mechanisms. Our results suggested that Trx1 may be a marker of HP with high pathogenicity and may play a pathogenic role by participating in the IL6/STAT3 pathway and inducing much more DNA damage. These results may provide a theoretical basis for the study of HP pathogenesis and benefit the clinical treatment in the future.

## Figures and Tables

**Table 1:** The display of primer sequences used in the research.

Primer	Primer sequence (5′ − 3′)
GAPDH (F)	AAATCAAGTGGGGCGATGCTG
GAPDH (R)	GCAGAGATGATGACCCTTTTG
HP Trx1 (F)	GGGGTTGCGTTAGTGGATTTTTG
HP Trx1 (R)	GACGACTTCGCCATCTTTTGTGA
16 s rRNA (F)	CCGCCTACGCGCTCTTTAC
16 s rRNA (R)	CTAACGAATAAGCACCGGCTAAC
IL6 (F)	AGGAGTGGCTAAGGACCAAGACC
IL6 (R)	TGCCGAGTAGACCTCATAGTGACC
OGG1 (F)	ATGGGGCATCGTACTCTAGC
OGG1 (R)	CTCCCTCCACCGGAAAGAT

## Data Availability

The data used to support the findings of this study are available from the corresponding author upon reasonable request.

## References

[1] Koper-Lenkiewicz O. M., Kaminska J., Gawronska B., Matowicka-Karna J. (2019). The role and diagnostic potential of gastrokine 1 in gastric cancer. *Cancer Management and Research*.

[2] Cover T. L., Blaser M. J. (2009). Helicobacter pylori in health and disease. *Gastroenterology*.

[3] Jiang J., Chen Y., Shi J., Song C., Zhang J., Wang K. (2016). Population attributable burden of Helicobacter pylori-related gastric cancer, coronary heart disease, and ischemic stroke in China. *European Journal of Clinical Microbiology & Infectious Diseases*.

[4] Hatakeyama M., Higashi H. (2005). Helicobacter pylori CagA: a new paradigm for bacterial carcinogenesis. *Cancer Science.*.

[5] Li Z., Lin S., Zhu J., Simao Y., Shigang D. (2000). Relationship between cagA gene and iceA gene of Helicobacter pylori and gastroduodenal diseases. *Journal of Peking University*.

[6] Lu J., Holmgren A. (2014). The thioredoxin antioxidant system. *Free Radical Biology & Medicine*.

[7] Holmgren A. (1985). Thioredoxin. *Annual Review of Biochemistry*.

[8] Shi Y., Liu L., Zhang T. (2013). The involvement of Helicobacter pylori thioredoxin-1 in gastric carcinogenesis. *Journal of Medical Microbiology*.

[9] L-n L., S-g D., Y-y S., H-j Z., Zhang J., Zhang C. (2016). Helicobacter pylori with high thioredoxin-1 expression promotes stomach carcinogenesis in Mongolian gerbils. *Clinics and Research In Hepatology And Gastroenterology*.

[10] Piao J. Y., Lee H. G., Kim S. J. (2016). Helicobacter pylori activates IL-6-STAT3 signaling in human gastric cancer cells: potential roles for reactive oxygen species. *Helicobacter*.

[11] Fathi N., Rashidi G., Khodadadi A., Shahi S., Sharifi S. (2018). STAT3 and apoptosis challenges in cancer. *International Journal of Biological Macromolecules*.

[12] Khashab F., Al-Saleh F., Al-Kandari N., Fadel F., Al-Maghrebi M. (2021). JAK inhibition prevents DNA damage and apoptosis in testicular ischemia-reperfusion injury via modulation of the ATM/ATR/Chk pathway. *International Journal of Molecular Sciences*.

[13] Guanizo A. C., Fernando C. D., Garama D. J., Gough D. J. (2018). STAT3: a multifaceted oncoprotein. *Growth Factors*.

[14] Johnson D. E., O'Keefe R. A., Grandis J. R. (2018). Targeting the IL-6/JAK/STAT3 signalling axis in cancer. *Nature Reviews. Clinical Oncology*.

[15] Menheniott T. R., Judd L. M., Giraud A. S. (2015). STAT3: a critical component in the response to helicobacter pylori infection. *Cellular Microbiology*.

[16] Yanyan S., Shigang D., Lu F., Jing Z., Linna L., Wong Y. (2011). Expression analysis of thioredoxin 1 of clinical strains of Helicobacter pylori in patients with gastric cancer and peptic ulcer. *Chinese Journal of Gastroenterology*.

[17] Shi Y., Wang P., Guo Y., Liang X., Li Y., Ding S. (2019). Helicobacter pylori-induced DNA damage is a potential driver for human gastric cancer AGS cells. *DNA and Cell Biology*.

[18] Yildirim Z., Bozkurt B., Ozol D. (2016). Increased exhaled 8-isoprostane and interleukin-6 in patients with Helicobacter pylori infection. *Helicobacter*.

[19] Baker L. M., Raudonikiene A., Hoffman P. S., Poole L. B. (2001). Essential thioredoxin-dependent peroxiredoxin system from Helicobacter pylori: genetic and kinetic characterization. *Journal of bacteriology.*.

[20] Fernandes A. P., Holmgren A. (2004). Glutaredoxins: glutathione-dependent redox enzymes with functions far beyond a simple thioredoxin backup system. *Antioxidants & Redox Signaling*.

[21] Lillig C. H., Berndt C., Holmgren A. (2008). Glutaredoxin systems. *Biochimica et Biophysica Acta*.

[22] Comtois S. L., Gidley M. D., Kelly D. J. (2003). Role of the thioredoxin system and the thiol-peroxidases Tpx and Bcp in mediating resistance to oxidative and nitrosative stress in Helicobacter pylori. *Microbiology (Reading, England)*.

[23] Filson H., Fox A., Kelleher D., Windle H. J., Sanders D. A. (2003). Purification, crystallization and preliminary X-ray analysis of an unusual thioredoxin from the gastric pathogen Helicobacter pylori. *Acta crystallographica Section D, Biological crystallography.*.

[24] Potamitou A., Holmgren A., Vlamis-Gardikas A. (2002). Protein levels of Escherichia coli thioredoxins and glutaredoxins and their relation to null mutants, growth phase, and function. *Journal of Biological Chemistry*.

[25] Lu J., Vlamis-Gardikas A., Kandasamy K. (2013). Inhibition of bacterial thioredoxin reductase: an antibiotic mechanism targeting bacteria lacking glutathione. *FASEB journal: official publication of the Federation of American Societies for Experimental Biology*.

[26] Nozawa R., Yokota T., Fujimoto T. (1989). Susceptibility of methicillin-resistant Staphylococcus aureus to the selenium-containing compound 2-phenyl-1,2-benzoisoselenazol-3(2H)-one (PZ51). *Antimicrobial Agents And Chemotherapy*.

[27] Farinati F., Cardin R., Bortolami M. (2008). Oxidative DNA damage in gastric cancer: CagA status and OGG1 gene polymorphism. *International Journal of Cancer*.

[28] Raza Y., Khan A., Farooqui A. (2014). Oxidative DNA damage as a potential early biomarker of Helicobacter pylori associated carcinogenesis. *Pathology & Oncology Research*.

[29] Shi Y. Y., Zhang J., Zhang T. (2018). Cellular stress and redox activity proteins are involved in gastric carcinogenesis associated with Helicobacter pylori infection expressing high levels of thioredoxin-1. *Journal of Zhejiang University. Science. B*.

[30] Shi Y. Y., Chen M., Zhang Y. X., Zhang J., Ding S. G. (2014). Expression of three essential antioxidants of Helicobacter pylori in clinical isolates. *Journal of Zhejiang University. Science. B*.

[31] Kumagae Y., Hirahashi M., Takizawa K. (2018). Overexpression of MTH1 and OGG1 proteins in ulcerative colitis-associated carcinogenesis. *Oncology Letters*.

[32] Fagard R., Metelev V., Souissi I., Baran-Marszak F. (2013). STAT3 inhibitors for cancer therapy: have all roads been explored?. *Jak-Stat*.

[33] Shih P. C. (2020). Revisiting the development of small molecular inhibitors that directly target the signal transducer and activator of transcription 3 (STAT3) domains. *Life Sciences*.

[34] Sekikawa A., Fukui H., Fujii S. (2005). REG Ialpha protein may function as a trophic and/or anti-apoptotic factor in the development of gastric cancer. *Gastroenterology*.

[35] Ji H. G., Piao J. Y., Kim S. J. (2016). Docosahexaenoic acid inhibitsHelicobacter pylori-induced STAT3 phosphorylation through activation of PPAR*γ*. *Molecular Nutrition & Food Research*.

[36] Linher-Melville K., Singh G. (2017). The complex roles of STAT3 and STAT5 in maintaining redox balance: lessons from STAT-mediated xCT expression in cancer cells. *Molecular and Cellular Endocrinology*.

[37] Busker S., Qian W., Haraldsson M. (2020). Irreversible TrxR1 inhibitors block STAT3 activity and induce cancer cell death. *Science Advances*.

[38] Kim N. H., Lee M. Y., Park S. J., Choi J. S., Oh M. K., Kim I. S. (2007). Auranofin blocks interleukin-6 signalling by inhibiting phosphorylation of JAK1 and STAT3. *Immunology*.

[39] Windle H. J., Fox A., Ni Eidhin D., Kelleher D. (2000). The thioredoxin system of Helicobacter pylori. *The Journal of Biological Chemistry*.

[40] Powis G., Montfort W. R. (2001). Properties and biological activities of thioredoxins. *Annual Review Of Biophysics And Biomolecular Structure*.

[41] Yu H., Lee H., Herrmann A., Buettner R., Jove R. (2014). Revisiting STAT3 signalling in cancer: new and unexpected biological functions. *Nature Reviews Cancer*.

